# Climate change diminishes the potential habitat of the bont tick (*Amblyomma hebraeum*): evidence from Mashonaland Central Province, Zimbabwe

**DOI:** 10.1186/s13071-022-05346-z

**Published:** 2022-06-28

**Authors:** Paradzayi Tagwireyi, Manuel Ndebele, Wilmot Chikurunhe

**Affiliations:** 1 Department of Geography, Geospatial Sciences and Earth Observation, University of Zimbabwe, 630 Churchill Road, Harare, Zimbabwe; 2 Department of Veterinary Services, 1327 Atherstone Road, Bindura, Zimbabwe

**Keywords:** Area under the curve (ROC), Climate change, Ensemble modelling, True skill statistic (TSS), Variance inflation factor (VIF)

## Abstract

**Background:**

Understanding the response of vector habitats to climate change is essential for vector management. Increasingly, there is fear that climate change may cause vectors to be more important for animal husbandry in the future. Therefore, knowledge about the current and future spatial distribution of vectors, including ticks (Ixodida), is progressively becoming more critical to animal disease control.

**Methods:**

Our study produced present (2018) and future (2050) bont tick (*Amblyomma hebraeum*) niche models for Mashonaland Central Province, Zimbabwe. Specifically, our approach used the Ensemble algorithm in Biomod2 package in R 3.4.4 with a suite of physical and anthropogenic covariates against the tick’s presence-only location data obtained from cattle dipping facilities.

**Results:**

Our models showed that currently (the year 2018) the bont tick potentially occurs in 17,008 km^2^, which is 60% of Mashonaland Central Province. However, the models showed that in the future (the year 2050), the bont tick will occur in 13,323 km^2^, which is 47% of Mashonaland Central Province. Thus, the models predicted an ~ 13% reduction in the potential habitat, about 3685 km^2^ of the study area. Temperature, elevation and rainfall were the most important variables explaining the present and future potential habitat of the bont tick.

**Conclusion:**

Results of our study are essential in informing programmes that seek to control the bont tick in Mashonaland Central Province, Zimbabwe and similar environments.

**Graphical Abstract:**

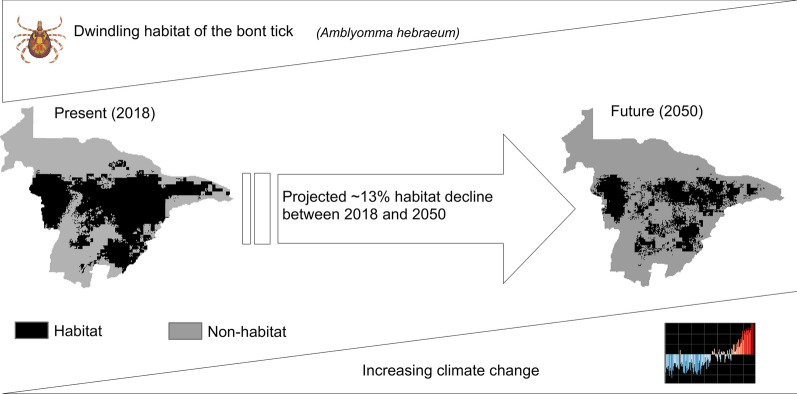

## Background

There is a notable increase in tick distribution and tick-borne diseases, particularly in the tropical and sub-tropical regions. Tick species of particular interest in the tropics include species in the genera *Hyalomma*, *Amblyomma* and *Rhipicephalus* [1, 2]. Some of these species, e.g., the bont tick (*Amblyomma hebraeum*), are challenging to control as they have very high reproductive rates and sometimes develop resistance to standard remedies. In addition, the bont tick has a wide spatial distribution because of its ability to survive in both dry and humid environments [3, 4]. Thus, the bont tick is a significant vector for various pathogens, including the obligate intracellular proteobacterium *Ehrlichia ruminantium*, which causes heartwater disease in ruminants [5].

Ticks spend much of their life away from their host. As such, ticks are prone to the effects of climatic and other environmental dynamics [6]. The predicted change in climate, primarily to warmer conditions, motivates the modelling of future distribution of ticks using climate change scenarios [7]. Those niche models use various environmental variables, including elevation temperature, rainfall and humidity [8]. Recently, Land Use Land Cover Change (LULCC) has been a significant covariate in modelling tick species and tick host distribution and is often used in modelling overlaps in space and time between human activities and ticks habitats [9].

The present and future distribution of ticks must be known to support vector management programmes [10]. However, while bont ticks are notorious for animal husbandry, their present and future spatial distribution and the drivers of that distribution are primarily unknown. This study used bont tick presence-only data and a suite of climate change scenarios data and environmental covariates to model the present and future distribution of one of the most notorious ticks in Mashonaland Central province, Zimbabwe. The objectives of this study were twofold: (1) identify the change in the potential habitat of bont tick between current time (2018) and future 2050 and (2) identify the drivers of the spatial distribution of bont tick. Results of this study are essential in informing programmes that seek to control the bont tick in Mashonaland Central Province, Zimbabwe, and similar environments.

## Methods

### Study site

**Fig. 1:**
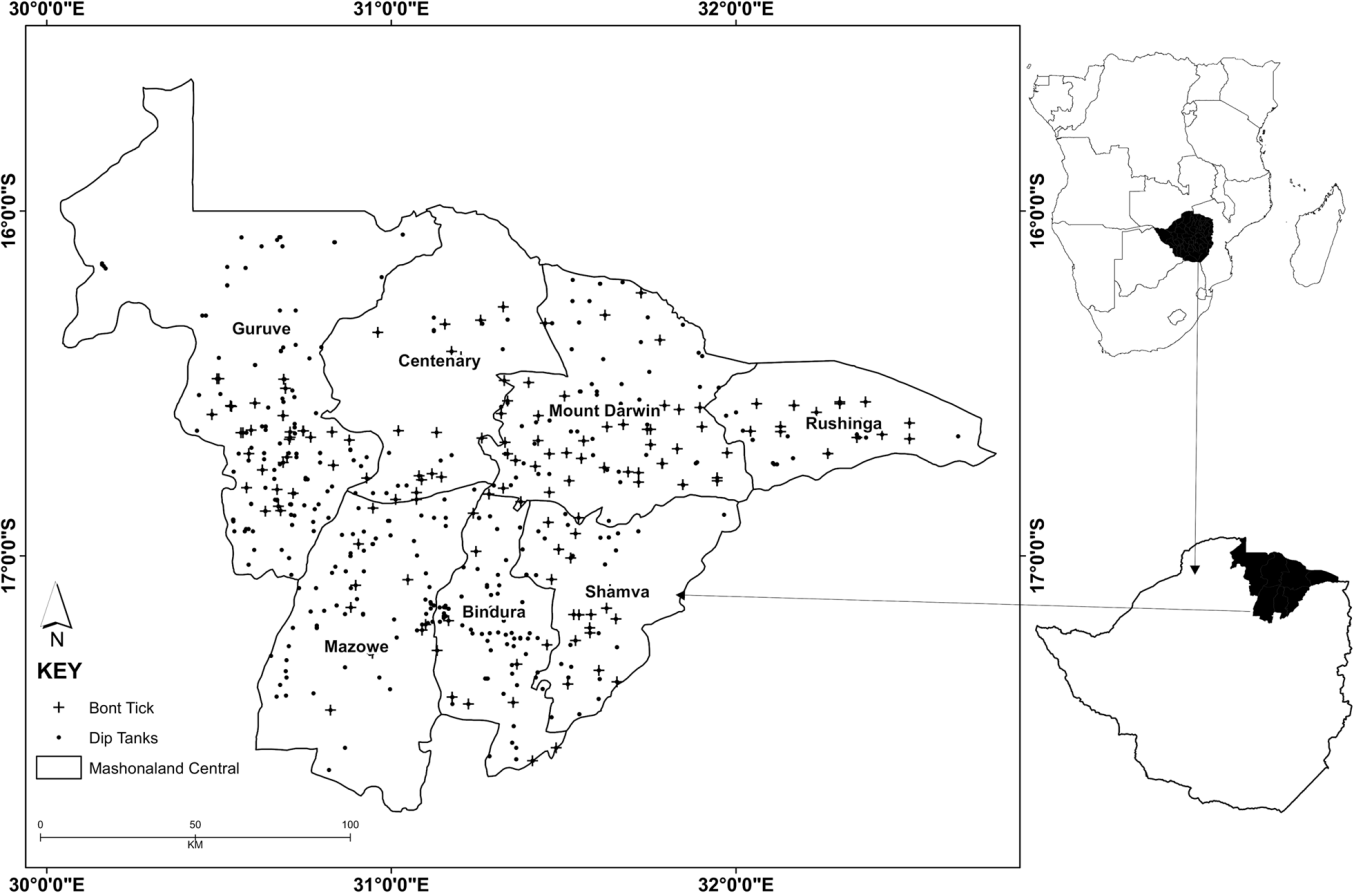
Location of Mashonaland Central Province in northeastern Zimbabwe showing the 270 presence-only bont tick and dip tank location data

The study area was Mashonaland Central Province, Zimbabwe, located between 30.04° E–32.75° E and 17.97° S–15.40° S, comprising 28,347 km^2^ of land (Fig. 1). According to the Zimbabwe National Census 2012 statistics, the province’s population was 1,152,520 inhabitants. A wide range of land tenure types coexists in the province, including communal areas, newly resettled small scale farming (A1), newly resettled large scale farming (A2), small-scale commercial farming, extensive-scale commercial farming and old resettlement areas [11]. The province comprises regions of varying agricultural potential, including Agro-ecological Region 2, which receives as much as 1000 mm of annual rainfall, to Agro-ecological Region 5, which receives < 450 mm of annual rainfall [12]. The area experiences a wet season (October to April) and a dry season (May to October).

Administratively, Mashonaland Province consists of eight districts: Bindura, Centenary, Mt Darwin, Guruve, Mbire, Rushinga, Shamva and Mazowe. Agriculture is the basis of the economy as the province is predominantly rural [13]. As such, the majority of the inhabitants of the province are small-holder farmers whose livelihoods depend on crops (e.g. maize, wheat, tobacco) and livestock (e.g. cattle, sheep, goats and poultry). However, cattle farming experiences economic losses due to tick-borne diseases, typical to resource-poor cattle owners in Africa [14].

Like other provinces in Zimbabwe, Mashonaland Central Province’s tick control is based on dipping cattle in plunge dips (concrete plunge tanks) containing acaricides diluted in large volumes of water. Farmers have to dip their cattle weekly during the wet season and fortnightly during the dry season. It has been the responsibility of the government to supply acaricides. However, the communal farmer pays a reasonable fee to the Department of Veterinary Services so that their herds of cattle benefit from the tick control exercise [14]. Dipping in acaricides has been a compulsory exercise since its legislation in 1914, and it has been one of the successful methods of tick control in livestock [15].

### Tick species geographical data

Veterinary extension officers collected ticks at all the cattle dipping facilities for which location coordinates were known. The procedure of getting the ticks involves manually restraining every tick-infested animal at the dipping facility to allow the physical examination. The veterinary officers used the morphological identification method to identified the tick species following [16] plus guides including [17] and completed tick inspection forms using morphology. The veterinary officers are experts in tick species identification. Since cattle dipping is done weekly during the wet season and fortnightly during the dry season, veterinary officers inspected cattle for ticks and recorded tick presence data every month from 2010. The tick species collected for the 2018 year of study were *Rhipicephalus decoloratus* (African blue tick), *Rhipicephalus sanguineus* (s.l.) (brown dog tick), *Rhipicephalus evertsi* (red-legged tick) and *Amblyomma hebraeum* (bont tick).

### Predictor variables

We used bioclimatic data obtained from the WorldClim Version 2 Server for both present (2018) and future (2050) predictions [18]. Specifically, we used the CCSM4 climate change scenario rcp45 with a spatial resolution of 2.5 min (Table 1).

**Table 1:** All Predictor variables before multicollinearity test

Code	Variable
BIO1	Annual mean temperature
B102	Mean diurnal range
BIO3	Isothermality
BIO4	Temperature seasonality
BIO5	Maximum temperature of warmest month
BIO6	Min temperature of coldest month
BIO7	Temperature annual range
BIO8	Mean temperature of wettest quarter
BIO9	Mean temperature of driest quarter
BIO10	Mean temperature of warmest quarter
BIO11	Mean temperature of coldest quarter
BIO12	Annual precipitation
BIO13	Precipitation of wettest month
BIO14	Precipitation of driest month
BIO15	Precipitation seasonality
BIO16	Precipitation of wettest quarter
BIO17	Precipitation of driest quarter
BIO18	Precipitation of warmest quarter
BIO19	Precipitation of coldest quarter
DEM	Digital elevation model
LandC	Land cover

We obtained the topographic and landcover variables from the ESA Climate Change Initiative [19] (Table 1). To model LULCC, we used the Land Change Modeler of Terrset Geospatial Monitoring and Modelling System because of its capability to incorporate Markov-Chain [20]. To model future land use and cover change up to 2050, we used a first-order Markov-Chain, a stochastic model for quantitative land change detection [21]. In addition, the Earth Explorer Aster Global DEM provided the elevation covariate [22] (Table 1).

All predictor variables were obtained in raster formats and adjusted to 1-km resolution and converted to ASCII format using ArcGIS 10.5 (Environmental Systems Research Institute, Redlands, CA, USA).

### Variable selection

To deal with multicollinearity, we used the Variance Inflation Factor (VIF), where the covariate with a VIF > 10 was excluded in the modelling following Tagwireyi et al. [23]. The VIF was calculated in R using USDM package [24]. After excluding the collinear variables, we remained with ten for both the present and future modelling (Tables 2 and 3).

**Table 2:** Variables used for present (2018) distribution of the Bont tick

Code	Variable	Variance inflation factor
B102	Mean diurnal range	5.47
BIO3	Isothermality	6.00
BIO5	Maximum temperature of warmest month	7.40
BIO13	Precipitation of wettest month	3.79
BIO14	Precipitation of driest month	2.10
BIO15	Precipitation seasonality	8.65
BIO18	Precipitation of warmest quarter	1.46
BIO19	Precipitation of coldest quarter	5.30
DEM	Digital elevation model	1.07
LandC	Land cover	1.05

Only variables with variance inflation factor (VIF) < 10 were used in the modelling

**Table 3:** Variables used for future (2050) distribution of the bont tick

Code	Variable	Variance inflation factor
B102	Mean diurnal range	5.18
BIO3	Isothermality	5.62
BIO5	Maximum temperature of warmest month	7.21
BIO13	Precipitation of wettest month	3.81
BIO14	Precipitation of driest month	2.01
BIO15	Precipitation seasonality	8.27
BIO18	Precipitation of warmest quarter	1.45
BIO19	Precipitation of coldest quarter	5.13
DEM	Digital elevation model	1.03
LandC	Land cover	1.05

Only variables with variance inflation factor (VIF) < 10 were used in the modelling

### Modelling procedure

The Ensemble algorithm in Biomod2 package was used for the modelling in R 3.4.4 [25]. The approach selected eight modelling techniques in fitting and averaging present and future species habitat predictions. These are generalized linear models (GLM), classification tree analysis (CTA) artificial neural networks (ANN), surface range envelope (SRE), generalized boosting model (GBM), Breiman and Cutler’s random forest for classification and regression (RF), mixture discriminant analysis (MDA) and multiple adaptive regression splines (MARS).

The choice of eight modelling techniques was based on each model's efficiency, especially in the evaluation of model response curves [26]. The default settings in all chosen modelling techniques were used because default settings usually come enhanced for species distribution models (SDMs) [27]. All models used a maximum of 100 iterations [26]. Both present and future prediction models were created using 80% of occurrences and pseudo-absence data; the remaining 20% was used for model evaluation. Binary maps were then used to classify maps as suitable or unsuitable following Muhoyi et al. [28].

### Model evaluation

The partial receiving operating characteristic (partial ROC) [29] was for validating our Ensemble models by making use of area under curve (AUC) analysis and true skill statistics (TSS), which considers omission and commission errors [30]. The TSS ranges from 0.0 to 1.0. A TSS value in range 0–0.5 was considered a poor model fit, 0.6 to 0.8 an acceptable fit and valid; any values > 0.8 were considered good to excellent [31]. The AUC criteria considered a model with values from 0.0 to 0.6 as inferior, 0.6–0.9 as useful and > 0.9 as excellent following Ndaimani et al. [32]. A TSS threshold of 0.6 was used to select models for the Ensemble model, following Gallien et al. [33].

### Statistical analysis

The suitable area was calculated for both present and future predictions from binary maps using the raster zonal geometry calculation function in ArcGIS 10.5. To assess whether there is a reduction or increase in the suitable habitat, a two-sample test for equal proportions using a chi-square test was executed.

## Results

The Ensemble model achieved excellent success in predicting the current habitat of the bont tick (TSS = 0.900, ROC = 0.979). In addition, the approach was also excellent at predicting the future potential distribution of bont tick (TSS = 0.898, ROC = 0.976).

The variable importance analysis showed that elevation and maximum temperature of the warmest month, precipitation of the warmest month and precipitation of the wettest month are variables with high importance in explaining the occurrences of bont ticks (Tables 4 and 5). For example, the species habitat suitability is high in areas with moderate elevation ranging from 800 to 1000 m above sea level and high in areas with moderate temperatures ranging from 26 to 29 °C. Conversely, land cover, precipitation of coldest quarter and isothermality have the lowest contribution in determining bont tick habitat for present and future predictions.

**Table 4:** Variable importance for the present (2018) distribution model

Code	Variable	Variable contribution
B102	Mean diurnal range	0.10
BIO3	Isothermality	0.02
BIO5	Maximum temperature of warmest month	0.35
BIO13	Precipitation of wettest month	0.27
BIO14	Precipitation of driest month	0.00
BIO15	Precipitation seasonality	0.15
BIO18	Precipitation of warmest quarter	0.22
BIO19	Precipitation of coldest quarter	0.05
DEM	Digital elevation model	0.36
LandC	Land cover	0.08

**Table 5:** Variable importance for the future (2050) distribution model

Code	Variable	Variable contribution
B102	Mean diurnal range	0.08
BIO3	Isothermality	0.02
BIO5	Maximum temperature of warmest month	0.20
BIO13	Precipitation of wettest month	0.23
BIO14	Precipitation of driest month	0.00
BIO15	Precipitation seasonality	0.07
BIO18	Precipitation of warmest quarter	0.26
BIO19	Precipitation of coldest quarter	0.03
DEM	Digital elevation model	0.19
LandC	Land cover	0.06

**Fig. 2:**
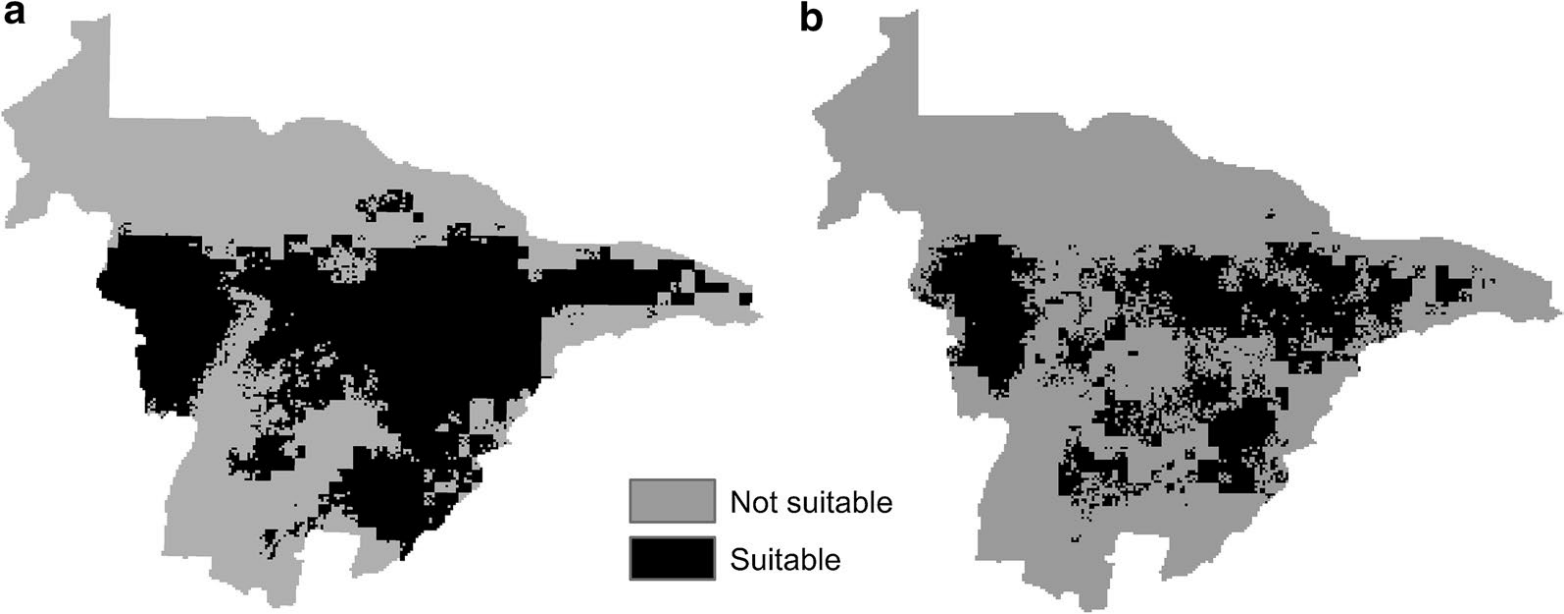
Binary maps of **a** current (2018) and **b** future (2050) potential distribution of bont tick in Mashonaland Central Province, Zimbabwe

Binary maps using the TSS = 0.6 thresholds showed the suitable habitat to be 17,008 km^2^ for the present model and 13,323 km^2^ for the future model (Fig. 2a, b), representing a decrease of bont tick potential habitat of ~ 13% (3685 km^2^) for Mashonaland Central Province, Zimbabwe.

The proportion of suitable habitat for the present model was 0.60, while that for the future model was 0.47. The chi-square test showed a significant difference between the present and future habitat sizes (*χ*^2^ = 3.40, df = 1, *P* = 2E−16).

## Discussion

We observed that three covariates, i.e., rainfall, elevation and temperature, are essential in explaining the predicted spatial distribution of the bont tick for both the present (2018) and future (2050). These three covariates directly affect the living conditions of tick species and tick hosts [34]. The potential occurrences of bont ticks were high in moderate climatic conditions and moderate elevation. However, a combination of very high temperatures and high elevation and a combination of very low elevation and very low temperature did not favour the occurrence of the bont tick.

Based on the prediction results, there would be a significant ~ 13% reduction in bont tick potential habitat by 2050. The mechanisms explaining the decline include an increase in temperature, which leads to saturation deficit, a thermodynamic factor that has an inverse relationship with temperature [35]. Ticks spend part of their life cycle stage of their time below the ground surface. Therefore, below ground conditions are critical for their survival [36]. Bont ticks prefer moderate ground conditions, neither too cold nor too hot. As such, the ~ 13% reduction of bont tick habitat by 2050 reflects a strong association between climate change and tick phenology [37].

Current studies and literature suggest land cover-land use as one of the essential factors in predicting the potential distribution of tick species [1, 2]. However, in our models, land cover and land use were not essential in explaining the potential distribution of bont tick for present and future predictions. Therefore, we speculate that land use and land cover change in our study area is insignificant to influence the spatial distribution of the bont tick.

**Fig. 3:**
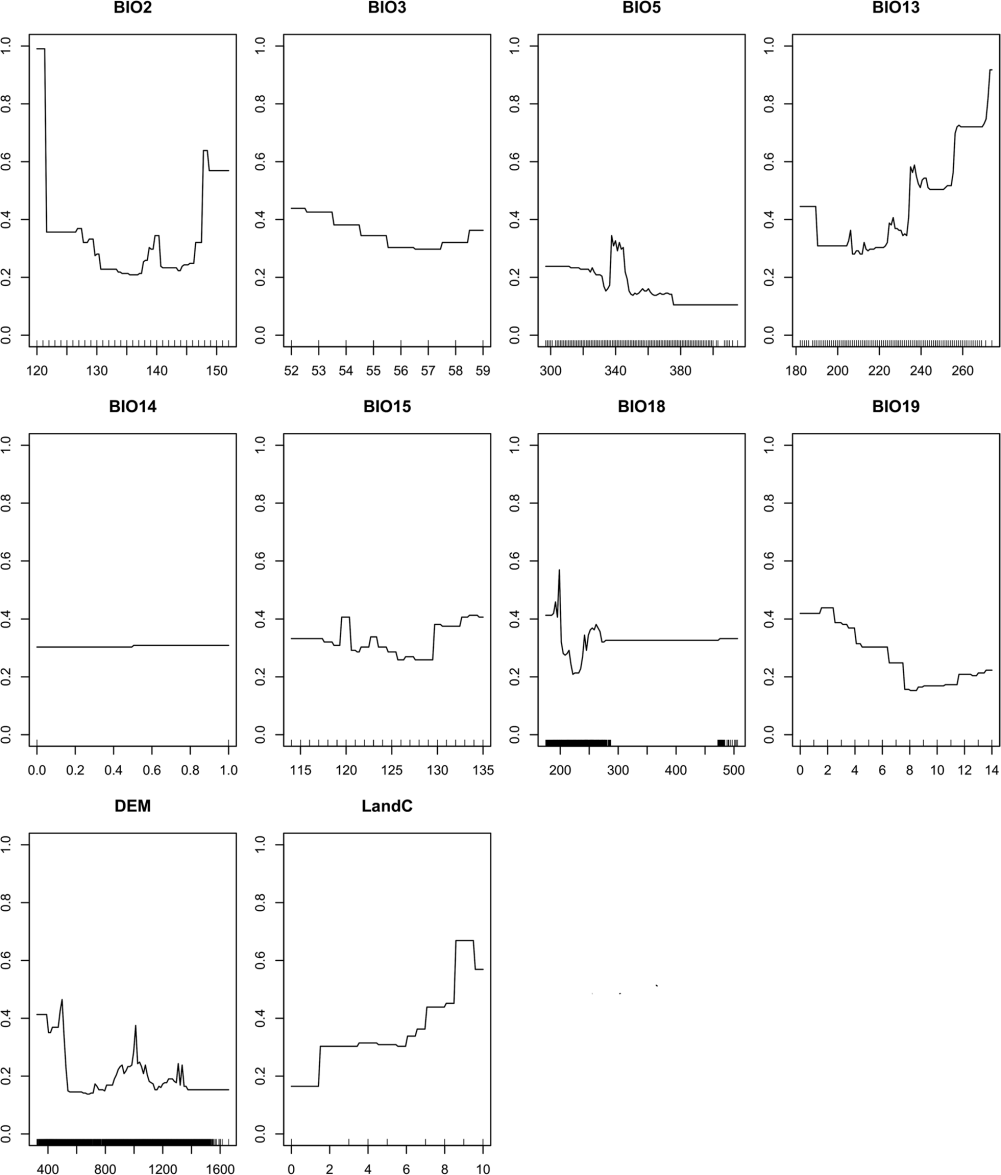
Variable response curves for the current (2018) potential distribution of bont tick in Mashonaland Central Province, Zimbabwe

**Fig. 4:**
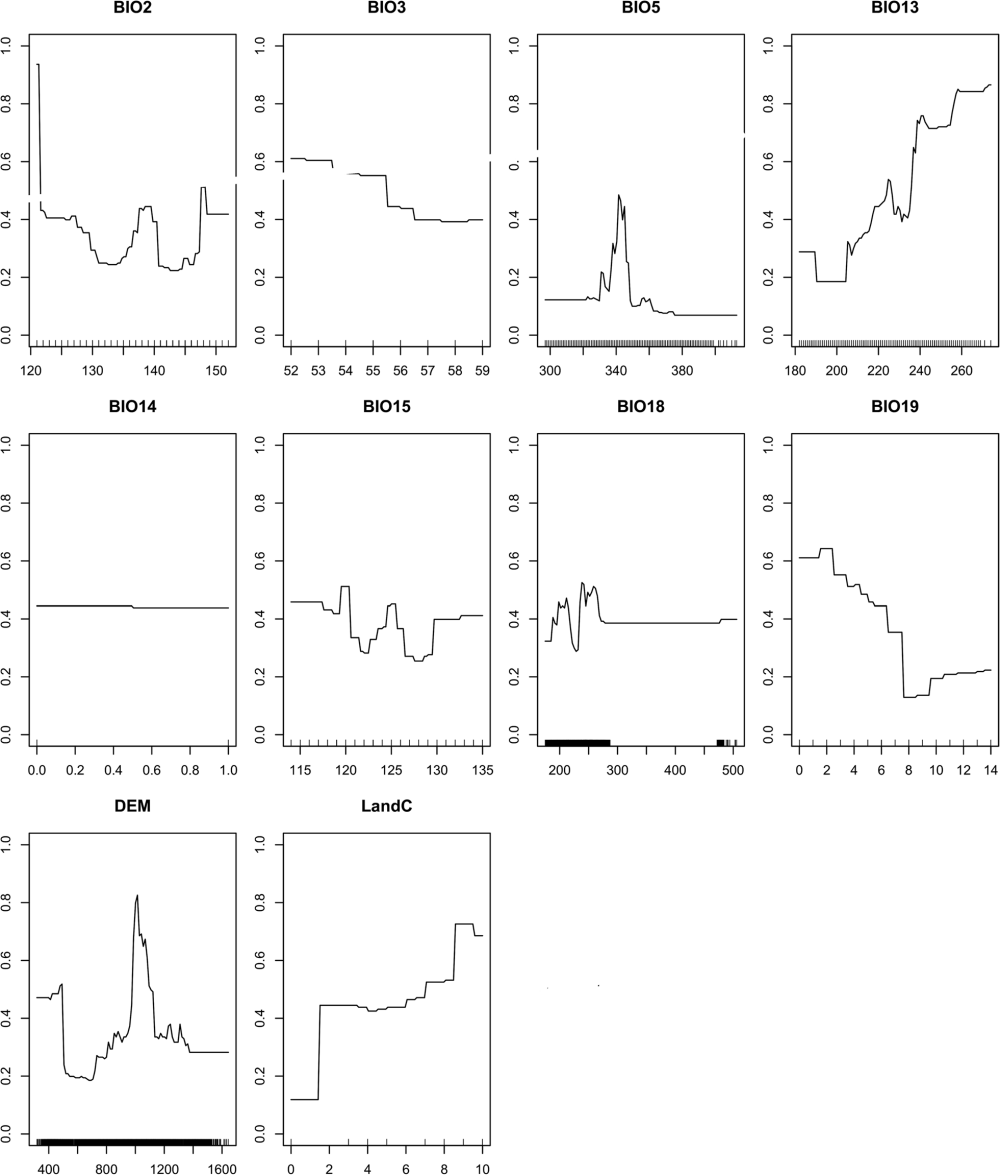
Variable response curves for the future (2050) potential distribution of bont tick in Mashonaland Central Province, Zimbabwe

The response of probability of the presence of the bont tick against the covariates for the present and the future models had very few differences. The models showed an increase in temperature (BIO5) leading to a decrease in the probability of bont tick present, with high sensitivity at around 26 °C to 29 °C (Figs. 3 and 4). Analysis of the elevation response curve showed high sensitivity response at moderate elevation (Fig. 3), with increases in altitude leading to a low probability of bont tick presence. Since elevation and temperature are related directly, the exact mechanisms of the saturation deficit suggested by Herrmann and Gern [37] could apply.

The weakness of our study is onefold. Using only cattle controlling points for the tick presence data technically excludes ticks not attached to cattle in the study. Future researchers could improve the modelling process by using other data collection methods with more comprehensive sample coverage than our study. However, our results are useful because of two major strengths of our approach. The first strength is our use of authenticated bioclimatic variables that have global spatial coverage. Thus, the variables are available to other scientists to validate our findings [38]. Second, our models maintain high validity and reliability by using the Ensemble model, which extracted the best of eight models [39]. As such, the results of our study are essential in informing programmes that seek to control the bont tick in Mashonaland Central Province, Zimbabwe, and similar environments.

## Conclusion

We successfully evaluated the association between environmental variables and bont tick occurrences in Mashonaland Central Province, Zimbabwe. We observed that the most critical environmental covariates which drive bont tick distribution are temperature, rainfall and elevation. We also successfully predicted both present (2018) and future (2050) bont tick potential habitat and observed that there would be a huge reduction of the potential habitat of the ticks by 2050. The study, however, could not include other tick host species in the modelling process; neither was it able to include tick samples outside tick control points. Therefore, we recommend considering tick data in all species that offer a host to bont tick as well as considering the abundance of those species in future work. This study provides baseline information for long-term tick eradication programmes.

## Data Availability

The *Amblyomma hebraeum* presence only raw data used in the study is published at https://figshare.com/articles/dataset/Climate_change_diminishes_the_potential_habitat_of_the_bont_tick_Amblyomma_hebraeum_evidence_from_Mashonaland_Central_Province_Zimbabwe_Raw_data/19816729.
